# Home based cardiac rehabilitation: A retrospective cohort analysis on all-cause mortality and hospital readmission rates across sexes and races

**DOI:** 10.1016/j.ajpc.2024.100708

**Published:** 2024-07-17

**Authors:** Zhengran Wang, Rachid Elkoustaf, Columbus Batiste, Debora Lahti, Janis F. Yao, Tadashi Funahashi

**Affiliations:** a Susan Samueli Integrative Health Institute, University of California, Irvine, Irvine, CA, USA; b Division of Cardiology, University of California, Irvine School of Medicine, Irvine CA, USA; c Southern California Permanente Medical Group, Pasadena, CA, USA; d Kaiser Permanente Center for Health Innovation, Tustin, CA, USA; e Kaiser Permanente Department of Research and Evaluation, Pasadena, CA, USA

**Keywords:** Cardiac rehab, Home-based cardiac rehab, HBCR, Preventive Cardiology

## Abstract

**Objective:**

Studies have shown that both home-based cardiac rehabilitation (HBCR) and center-based cardiac rehabilitation (CBCR) exhibit comparable efficacy in reducing mortality during short-term follow-up periods of up to 12 months. However, research on sex- and race-specific outcomes associated with HBCR is limited. This study examines all-cause mortality and hospital readmission among patients referred to HBCR, with stratification by sex and race.

**Methods:**

This Kaiser Permanente Southern California (KPSC) retrospective cohort study followed 6,868 patients from HBCR referral until death, disenrollment, or December 31, 2021. There were 3,835 HBCR graduates, 722 non-graduates, and 2,311 non-enrolled patients. Cox models were used to estimate hazard ratios (HR) and 95 % confidence intervals (CI) comparing 1) HBCR graduates vs. non-enrolled, and 2) HBCR graduates vs. non-graduates, stratified by sex and race. Differential outcomes among strata were analyzed using Kaplan-Meier curves.

**Results:**

Among the 6,868 patients referred to HBCR, 4693 (68.3 %) were male, 2,175 (31.7 %) female, 870 (12.7 %) Asian/Pacific Islander, 731 (10.6 %) African American, 1,612 (23.6 %) Hispanic/Latino, and 3,646 non-Hispanic White (53.1 %). Over a mean follow-up period of 2.28 years, HBCR graduates, compared to patients who did not enroll in HBCR, had overall significantly lower risks of all-cause mortality and hospitalization. These results remained significant with stratification by sex and race. Compared to HBCR non-graduates, HBCR graduates overall had significantly lower risks of all-cause mortality and hospitalization. In the same comparison, mortality risk was significantly reduced for male and White patients; risk of hospital readmission was significantly reduced in both sexes, African American, and White patients. Among HBCR graduates, no significant differences in all-cause mortality or hospital readmission were observed across sexes and races.

**Conclusion:**

HBCR participation is associated with reduction of all-cause mortality and hospital readmission rates across sexes and races. Notably, we observed benefits at varying levels of engagement, which suggests that even partial completion of HBCR is associated with risk reduction. Among HBCR graduates, we found similar outcomes across sexes and races, which suggests that the program can be effective among diverse patient groups.

## Highlights

• HBCR program completion significantly lowers all-cause mortality and hospital readmission rates.
• The benefits of HBCR program completion persist across sexes and races without differential outcomes.
• A dose-response relationship between participation in the HBCR program and health outcome is evident in certain patient groups.

## Graphical abstract

**Figure:**
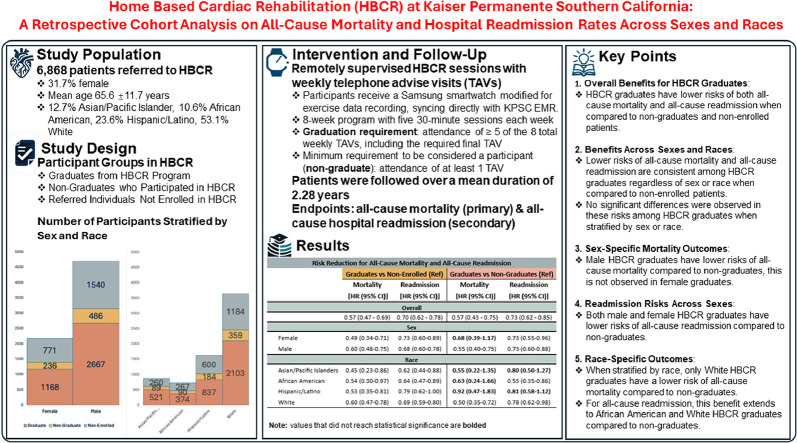

## 1 Introduction

Cardiac rehabilitation (CR) is a well-validated strategy for secondary prevention of cardiovascular diseases. It has been shown to reduce mortality, hospital readmission, and recurrent adverse cardiac events [[1], [2], [3], [4], [5]]. First introduced in the 1960s, CR is traditionally carried out in the center-based setting [5,6]. Despite its effectiveness, the utilization of CR has historically been low. This continues to be the case due to the presence of various barriers, such as accessibility issues, scheduling conflicts, and high costs. Over the past few decades, U.S. data from various sources has indicated that the referral rate for CR ranged from 40 % to 81 %. Participation rates have been even lower. In 2016, the enrollment rate for Medicare patients was reportedly 24 % [7,8]. As an alternative to CBCR, HBCR was developed in 1980s to help address the barrier of accessibility [9]. Evidence from numerous studies has demonstrated the non-inferiority of HBCR relative to the conventional CBCR in achieving significant reductions in mortality, enhancing exercise capacity, and favorably modifying cardiovascular risk factors [5,[10], [11], [12], [13], [14]]. Furthermore, HBCR has demonstrated superiority over CBCR in curbing hospital readmissions within the initial 12-month period post-intervention [15]. However, it's important to recognize the limitations of these findings, as most HBCR studies had relatively short-term follow-up periods of 12 months or less [5,[10], [11], [12], [13], [14], [15]]. The demand for remote healthcare delivery models has significantly increased since the COVID-19 pandemic, leading to a surge in demand for HBCR in line with this trend [6]. As HBCR becomes increasingly important in the field of CR, there is a clear need to strengthen the scientific foundation supporting this modality. The lack of studies with longer follow-up duration presents a significant gap that requires attention. A thorough understanding of the long-term effectiveness and sustainability of benefits of HBCR is crucial for ensuring its proper implementation to improve patient outcomes.

At the same time, addressing the underrepresentation and underutilization of CR among women and racial minorities is essential [8]. Similarly, there is limited representation of females and racial minorities in the current HBCR literature [5]. These issues are concerning because females and certain racial minorities are disproportionately affected by cardiovascular diseases and could potentially benefit from CR [[16], [17], [18]].

To shed light on the above issues, we conducted a survival analysis over a 3.75-year follow up period, examining a diverse group of patients of KPSC who were referred to HBCR. With stratification by sex and race, we compared the survival and hospital readmission rates of HBCR graduates to HBCR non-graduates and non-enrolled patients, respectively.

## 2 Methods

KPSC is an integrated health care delivery system with approximately 4.8 million members within a service area comprising >20 % of Southern California's population. KPSC membership is diverse and widely representative of the Southern California region [19]. Members’ receipt of outpatient, inpatient, laboratory, and pharmacy services are tracked in the electronic health record (EHR) system. Services performed outside of KPSC are tracked through submitted billing claims. The institutional review board at KPSC reviewed and approved the current study and a waiver of informed consent was granted due to the data-only nature of the study. The study adhered to the reporting guideline known as Strengthening the Reporting of Observational Studies in Epidemiology (STROBE).

This study was conducted at 12 centers across KPSC. At KPSC, all patients with the appropriate clinical indications are eligible for CR referral [20]. The decision for referral is at the discretion of the treating physician. We retrospectively identified all KPSC patients over the age of 18 who received a referral for HBCR between March 1, 2018, and May 1, 2020 for initial screening. Patients who did not have binary sex categorization (i.e. male or female), and those whose race were not categorized as Asian/Pacific Islander, Hispanic Latino, African American, or non-Hispanic White (Hereafter White) were not included in the study. A total of 7474 patients were eligible to enter initial screening. Among these patients, 4928 (65.9 %) enrolled in HBCR and 2546 (34.1 %) did not. We subsequently excluded patients without continuous membership with KPSC for at least 365 days prior to HBCR enrollment, with a permissible gap in membership of up to 60 days. Among the 4928 enrolled patients, 4557 (92.3 %) met the membership criterion and were included in the final analytic sample. Among the 4557 patients, 3835 (84.2 %) graduated from HBCR while 722 (15.8 %) only participated but did not meet graduation requirement. Among the 2546 non-enrolled patients, 2311 (90.8 %) met the membership criterion and were included in the final analytic sample. A total of 6868 patients were included in the final analytic sample. The enrollment period for HBCR was from April 3, 2018, to June 30, 2020.

The HBCR program at KPSC is an 8-week remotely supervised home exercise program with 5 sessions per week, each lasting 30 min. Participants are provided with a modified version of Samsung smartwatch, which records data on exercise and forwards it to KPSC EMR. A HeatWise mobile application is also provided, which is used to log exercise, medications, and symptoms. Participants receive a weekly call known as the Telephone Advice Visit (TAV) from a CR nursing staff for progress tracking. Health education classes, behavioral coaching, depression screening, and psychological support are also integral parts of the program. *Funahashi* et al. have previously outlined the specifics of the HBCR program at KPSC [21]. Among patients who enrolled in HBCR, participation was defined as attendance of ≥ 1 TAV, and graduation was defined as attendance of ≥ 5 of the 8 total weekly TAVs, including the required final TAV.

The primary outcome of interest included all-cause mortality, defined as death from any cause occurring on or prior to December 31, 2019. The secondary outcome of interest was all-cause hospitalization. For the current study, individuals were followed using electronic health records from referral to HBCR until death, disenrollment, or administrative censoring on December 31, 2021.

Demographics and clinical characteristics included age, sex (i.e. male or female), race (i.e. Asian/Pacific Islander, Hispanic Latino, African American, or White), and body mass index (BMI). Baseline comorbidities included coronary artery disease (CAD), stable angina, hypertension, hyperlipidemia, chronic heart failure (HF), prior myocardial infarction (MI), peripheral vascular disease (PVD), family history of CAD, diabetes, cancer, smoking status (i.e., current vs. former use), obesity, chronic kidney disease (CKD) with staging, and end-stage renal disease (ESRD). In addition, the Healthy Places Index® (HPI) of each patient was categorized into quartiles. In brief, the HPI is a weighted score that aims to comprehensively assess the well-being of patients residing in different communities [22].

Prior cardiovascular procedures, including coronary artery bypass graft (CABG), percutaneous coronary intervention (PCI), heart valve repair, were also identified. All diagnoses were identified in KPSC EMR using The International Classification of Diseases 10th Revision, Clinical Modification (ICD-10-CM) codes. All procedures were identified using Current Procedural Terminology (CPT) codes.

Select adjuvant pharmacotherapies were identified based on outpatient prescription refills, which included aspirin, thienopyridines, coumadin, heparin, thrombin inhibitors, factor Xa inhibitors, statins, angiotensin converting enzyme (ACE) inhibitors, and beta blockers. The number of health education classes attended at the KPSC Center for Healthy Living (CHL) and the number of TAVs attended were also collected.

Comparisons of baseline continuous variables and categorical variables across sexes and races were performed using Kruskal-Wallis test and χ^2^ test, respectively. The primary endpoint of the study is all-cause mortality over the 3.75-year study period. The secondary endpoint is all-cause hospital readmission over the same period. The study period spans from the first enrollment date of April 3, 2018, to December 31, 2021, the final date of follow up. For each patient, living status and hospital readmissions, if any, during this period was retrospectively identified in KPSC EMR. To assess the effectiveness of HBCR in reducing mortality and hospital readmission within each sex or race, we conducted survival analyses within each category of patients. Hazard ratios (HRs) were calculated for the following comparisons: HBCR graduates versus non-enrolled patients, and HBCR graduates versus non-graduates. Log rank tests were used to detect differences between groups compared. The multivariate Cox proportional hazard model was used to examine whether the hazard ratio of interest would change significantly, while controlling for other potential covariates. Covariates in the Cox model were selected by clinical judgment. In addition to graduation status, other covariates include age, BMI group, HPI divided into quartiles, race, diabetes, hypertension, cancer, hyperlipidemia, former smoking, current smoking, family history of coronary artery disease, CKD of any stage, ESRD, history of acute MI, stable angina, stable HF, CAD, CABG, PCI, heart valve repair, ACE inhibitor use, aspirin use, beta blocker use, factor Xa inhibitor use, thienopyridine use, thrombin inhibitor use, statin use, and warfarin use. A sensitivity analysis using propensity adjustment was conducted to validate the robustness of the original multivariate analysis. To compare the effectiveness of HBCR in reducing mortality and hospital readmission across sexes and races, we also conducted survival analyses for HBCR graduates between sexes and among races.

Missing data were observed in race and HPI categories. Patients whose race data was missing or categorized as “other” were excluded from the study. Patients with missing HPI data were pooled into a separate group, which was used in multivariate analysis along with the four quartile groups with known HPI.

All p-values were two-sided and a *p* < 0.05 was considered statistically significant. All analyses were conducted using SAS Enterprise Guide version 8.2 (Cary, NC)

## 3 Results

**Fig. 1:**
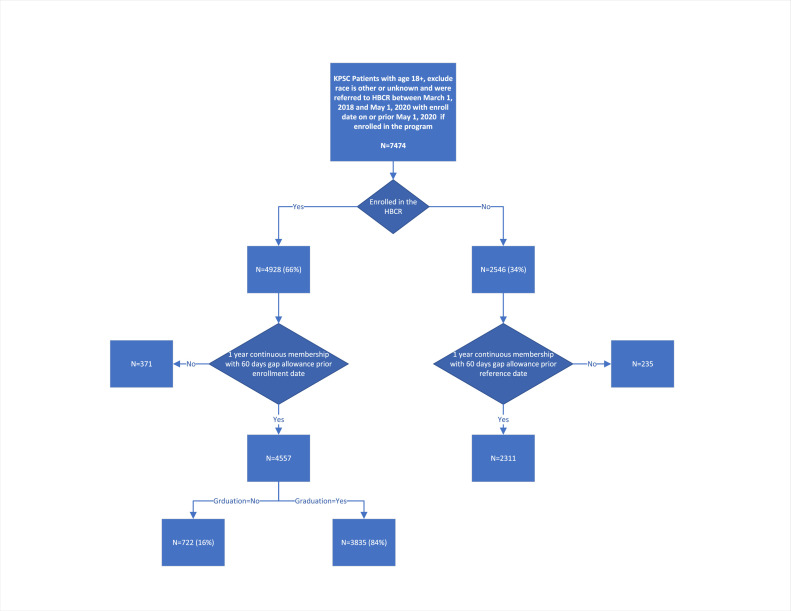
Selection process of the study.

A total of 7474 patients who received HBCR referrals were identified through initial screening, among which 4928 (65.9 %) were enrolled in HBCR and 2546 (34.1 %) did not enroll. Based on the membership criterion, a total of 6868 (91.9 %) referred patients were included in the study, of which 4557 (92.5 %) enrolled in HBCR and 2311 (90.8 %) did not enroll (Fig. 1). Among patients who enrolled in HBCR, 3835 (84.2 %) graduated and 722 (15.8 %) did not graduate. The maximum duration of follow-up period was 3.75 years, and the minimum was 0.02 years due to patient death. The average follow-up period was 2.28 years. Detailed baseline patient characteristics are outlined in Table 1. Among HBCR graduates, non-graduates, and non-enrolled patients, differences in baseline characteristics were observed in multiple categories.

**Table 1:** Baseline Characteristics.

	HBCR Participation Status				
	Graduate (*N* = 3835)	Non-Graduate (*N* = 722)	Non-Enrolled (*N* = 2311)	Total (*N* = 6868)	P-value
**Sex, n (%)**					0.0494^1^
Female n (%)	1168 (30.5 %)	236 (32.7 %)	771 (33.4 %)	2175 (31.7 %)	
Male n (%)	2667 (69.5 %)	486 (67.3 %)	1540 (66.6 %)	4693 (68.3 %)	
**Age**					<0.0001^2^
Mean (SD)	64.5 (11.3)	66.3 (11.9)	67.2 (12.1)	65.6 (11.7)	
Median (IQR)	65 (57–72)	67 (59–74)	69 (59–76)	67 (58–74)	
Range	18–99	20–96	19–96	18–99	
**Age Group, n (%)**					<0.0001^1^
18–49	387 (10.1 %)	68 (9.4 %)	200 (8.7 %)	655 (9.5 %)	
50–59	792 (20.7 %)	128 (17.7 %)	378 (16.4 %)	1298 (18.9 %)	
60–69	1292 (33.7 %)	220 (30.5 %)	636 (27.5 %)	2148 (31.3 %)	
70–79	1072 (28.0 %)	214 (29.6 %)	755 (32.7 %)	2041 (29.7 %)	
80+	292 (7.6 %)	92 (12.7 %)	342 (14.8 %)	726 (10.6 %)	
**BMI**					0.0158^2^
Mean (SD)	30.2 (6.1)	30.1 (6.2)	29.7 (6.5)	30.0 (6.3)	
Median (IQR)	29.1 (26.0–33.5)	29.3 (25.8–33.5)	28.8 (25.2–33.3)	29.0 (25.7–33.4)	
Range	16.3–71.8	17.4–61.0	14.4–74.9	14.4–74.9	
**BMI Group, n (%)**					0.0001^1^
Underweight	26 (0.7 %)	4 (0.6 %)	30 (1.3 %)	60 (0.9 %)	
Normal Weight	686 (17.9 %)	141 (19.5 %)	513 (22.2 %)	1340 (19.5 %)	
Overweight	1454 (37.9 %)	254 (35.2 %)	796 (34.4 %)	2504 (36.5 %)	
Obese	1669 (43.5 %)	323 (44.7 %)	972 (42.1 %)	2964 (43.2 %)	
**Race, n (%)**					<0.0001^1^
Asian/Pacific Islanders	521 (13.6 %)	89 (12.3 %)	260 (11.3 %)	870 (12.7 %)	
African American	374 (9.8 %)	90 (12.5 %)	267 (11.6 %)	731 (10.6 %)	
Hispanic/Latino	837 (21.8 %)	184 (25.5 %)	600 (26.0 %)	1621 (23.6 %)	
White	2103 (54.8 %)	359 (49.7 %)	1184 (51.2 %)	3646 (53.1 %)	
**Healthy Places Index by Quartile, n (%)**					<0.0001^1^
1st (≤25 Percentile)	684 (18.1 %)	166 (23.2 %)	434 (23.7 %)	1284 (20.3 %)	
2nd (26–50 Percentile)	1151 (30.5 %)	224 (31.3 %)	561 (30.6 %)	1936 (30.6 %)	
3rd (51–75 Percentile)	1161 (30.7 %)	223 (31.1 %)	505 (27.5 %)	1889 (29.9 %)	
4th (76–100 Percentile)	780 (20.7 %)	103 (14.4 %)	334 (18.2 %)	1217 (19.2 %)	
Information Missing	59	6	477	542	
**Baseline Comorbidities, n (%)**					
Acute Myocardial Infarction, History of	719 (18.7 %)	157 (21.7 %)	459 (19.9 %)	1335 (19.4 %)	0.1434^1^
Cancer, History of	704 (18.4 %)	135 (18.7 %)	433 (18.7 %)	1272 (18.5 %)	0.9258^1^
Chronic Kidney Disease, Stage 1	46 (1.2 %)	9 (1.2 %)	37 (1.6 %)	92 (1.3 %)	0.4041^1^
Chronic Kidney Disease, Stage 2	284 (7.4 %)	62 (8.6 %)	202 (8.7 %)	548 (8.0 %)	0.1417^1^
Chronic Kidney Disease, Stage 3	653 (17.0 %)	147 (20.4 %)	514 (22.2 %)	1314 (19.1 %)	<0.0001^1^
Chronic Kidney Disease, Stage 4	80 (2.1 %)	31 (4.3 %)	125 (5.4 %)	236 (3.4 %)	<0.0001^1^
Chronic Kidney Disease, Stage 5	43 (1.1 %)	18 (2.5 %)	51 (2.2 %)	112 (1.6 %)	0.0008^1^
Chronic Kidney Disease, All Stages	904 (23.6 %)	212 (29.4 %)	705 (30.5 %)	1821 (26.5 %)	<0.0001^1^
Congestive Heart Failure, Stable	234 (6.1 %)	49 (6.8 %)	175 (7.6 %)	458 (6.7 %)	0.0809^1^
Coronary Artery Disease	2599 (67.8 %)	506 (70.1 %)	1593 (68.9 %)	4698 (68.4 %)	0.3770^1^
Diabetes	1578 (41.1 %)	359 (49.7 %)	1108 (47.9 %)	3045 (44.3 %)	<0.0001^1^
End Stage Renal Disease	93 (2.4 %)	29 (4.0 %)	113 (4.9 %)	235 (3.4 %)	<0.0001^1^
Family History of Coronary Artery Disease	1742 (45.4 %)	314 (43.5 %)	957 (41.4 %)	3013 (43.9 %)	0.0087^1^
Hyperlipidemia	3115 (81.2 %)	594 (82.3 %)	1861 (80.5 %)	5570 (81.1 %)	0.5545^1^
Hypertension	2613 (68.1 %)	528 (73.1 %)	1745 (75.5 %)	4886 (71.1 %)	<0.0001^1^
Morbid Obesity	946 (24.7 %)	210 (29.1 %)	577 (25.0 %)	1733 (25.2 %)	0.0404^1^
Smoking, Current	422 (11.0 %)	105 (14.5 %)	341 (14.8 %)	868 (12.6 %)	<0.0001^1^
Smoking, Former	1658 (43.2 %)	331 (45.8 %)	1081 (46.8 %)	3070 (44.7 %)	0.0208^1^
Stable Angina	597 (15.6 %)	125 (17.3 %)	401 (17.4 %)	1123 (16.4 %)	0.1420^1^
**Prior Cardiovascular Procedures, n (%)**					
Coronary Artery Bypass Graft	658 (17.2 %)	135 (18.7 %)	316 (13.7 %)	1109 (16.1 %)	0.0002^1^
Percutaneous Coronary Intervention	400 (10.4 %)	100 (13.9 %)	214 (9.3 %)	714 (10.4 %)	0.0020^1^
Heart Valve Repair	460 (12.0 %)	89 (12.3 %)	230 (10.0 %)	779 (11.3 %)	0.0341^1^
**Baseline Adjuvant Pharmacotherapies, n (%)**					
ACE Inhibitor	1462 (38.1 %)	271 (37.5 %)	980 (42.4 %)	2713 (39.5 %)	0.0021^1^
Aspirin	3114 (81.2 %)	593 (82.1 %)	1833 (79.3 %)	5540 (80.7 %)	0.1111^1^
Beta Blocker	3453 (90.0 %)	653 (90.4 %)	2081 (90.0 %)	6187 (90.1 %)	0.9435^1^
Factor Xa Inhibitor	109 (2.8 %)	15 (2.1 %)	51 (2.2 %)	175 (2.5 %)	0.2161^1^
Thienopyridine	2050 (53.5 %)	400 (55.4 %)	1229 (53.2 %)	3679 (53.6 %)	0.5669^1^
Thrombin Inhibitor	345 (9.0 %)	71 (9.8 %)	241 (10.4 %)	657 (9.6 %)	0.1750^1^
Statin	3482 (90.8 %)	656 (90.9 %)	2091 (90.5 %)	6229 (90.7 %)	0.9072^1^
Warfarin	596 (15.5 %)	119 (16.5 %)	345 (14.9 %)	1060 (15.4 %)	0.5788^1^
**Miscellaneous**					
**Telephone Advise Visits**					<0.0001^2^
Mean (SD)	6.3 (1.0)	2.1 (1.6)	N/A	5.7 (1.9)	
Median (IQR)	6.0 (6.0–7.0)	2.0 (0.0–4.0)	N/A	6.0 (5.0–7.0)	
Range	0.0–9.0	0.0–4.0	N/A	0.0–9.0	
**Center for Healthy Living Classes**					0.4482^2^
Mean (SD)	1.6 (1.4)	1.5 (1.5)	N/A	1.6 (1.4)	
Median (IQR)	1.0 (1.0–2.0)	1.0 (1.0–2.0)	N/A	1.0 (1.0–2.0)	
Range	0.0–11.0	0.0–11.0	N/A	0.0–11.0	

Abbreviations: SD = Standard Deviation; IQR = Interquartile Range; N/*A* = not applicable.

1 Chi-Square p-value;.

2 Kruskal-Wallis p-value;.

Among the 6868 patients included in the study, there were 4693 males (68.3 %) and 2175 females (31.7 %); 870 (12.7 %) Asian/Pacific Islanders, 731 (10.6 %) African American, 1621 (23.6 %) Hispanic/Latino, and 3646 (53.1 %) White. The mean age was 65.6 (standard deviation [SD] 11.7). The mean BMI was 30.0 (SD 6.3). Overall, 60 (0.9 %), 1340 (19.5 %), 2504 (36.5 %), and 2964 (43.2 %) were underweight, normal-weight, overweight, and obese, respectively. For HPI, 1284 (20.3 %), 1936 (30.6 %), 1889 (29.9 %), and 1217 (19.2 %) fell into to the first, second, third, and fourth quartile, respectively. The HPIs of 542 patients were missing. Hyperlipidemia (81.1 %), hypertension (71.1 %), CAD (68.4 %), former smoking (44.7 %), and diabetes (44.3 %) were the five most prevalent comorbidities. 1109 (16.1 %) had prior CABG, 714 (10.4 %) had prior PCI, and 779 (11.3 %) had prior valve replacement. Statins (90.7 %), beta blockers (90.1 %), aspirin (80.7 %), thienopyridines (53.6 %), and ACE inhibitors (39.5 %) were the most frequently prescribed five classes of medications. The HBCR graduates attended an average of 1.6 (SD 1.4) classes and 6.3 (SD 1.0) TAVs. In contrast, the HBCR non-graduates attended an average of 1.5 (SD 1.5) classes and 2.1 (SD 1.6) TAVs.

There were a total of 211 (5.5 %), 77 (10.7 %), and 286 (12.4 %) deaths among HBCR graduates, non-graduates, and non-enrolled patients, respectively. HBCR graduates had a lower multivariate-adjusted mortality hazard compared to non-enrolled patients (HR 0.57, 95 % CI 0.47–0.69, *P* < 0.0001). With stratification by sex, mortality hazards in graduates of both sexes were significantly reduced compared to non-enrolled patients. The results remained the same while adjusting for other covariates in multivariate analysis. With stratification by race, mortality hazards in graduates of all races were also significantly reduced compared to non-enrolled patients. Same results were observed in multivariate analysis (Table 2).

**Table 2:** All-cause mortality and hospital readmission.

	Graduates vs Non-Enrolled (Reference)		Graduates vs Non-Gradautes (Reference)	
	HR (95 % CI)	P-value^1^	HR (95 % CI)	P-value^1^
**Overall**				
**Mortality**				
Crude	0.44 (0.37 - 0.52)	<0.0001	0.47 (0.36 - 0.62)	<0.0001
Adjusted	0.57 (0.47 - 0.69)	<0.0001	0.57 (0.43 - 0.75)	<0.0001
**Readmission**				
Crude	0.63 (0.57 - 0.70)	<0.0001	0.64 (0.55 - 0.75)	<0.0001
Adjusted	0.70 (0.62 - 0.78)	<0.0001	0.73 (0.62 - 0.85)	<0.0001
**Sex**				
**Mortality - Female**				
Crude	0.41 (0.29 - 0.57)	<0.0001	0.51 (0.31 - 0.86)	0.0095
Adjusted	0.49 (0.34–0.71)	0.0001	**0.68 (0.39–1.17)**	**0.1638**
**Mortality - Male**				
Crude	0.45 (0.36 - 0.55)	<0.0001	0.46 (0.34 - 0.62)	<0.0001
Adjusted	0.60 (0.48–0.75)	<0.0001	0.55 (0.40–0.75)	0.0002
**Readmission - Female**				
Crude	0.64 (0.53 - 0.76)	<0.0001	0.65 (0.49 - 0.85)	0.0017
Adjusted	0.73 (0.60–0.89)	0.0018	0.73 (0.55–0.96)	0.0263
**Readmission - Male**				
Crude	0.62 (0.55 - 0.71)	<0.0001	0.64 (0.53 - 0.77)	<0.0001
Adjusted	0.68 (0.60–0.78)	<0.0001	0.73 (0.60–0.88)	0.0013
**Race**				
**Mortality - Asian/Pacific Islanders**				
Crude	0.37 (0.21 - 0.63)	0.0002	0.40 (0.18 - 0.86)	0.0159
Adjusted	0.45 (0.23–0.86)	0.0151	**0.55 (0.22–1.35)**	**0.1922**
**Mortality - African American**				
Crude	0.35 (0.21 - 0.59)	<0.0001	**0.55 (0.25 - 1.21)**	**0.1325**
Adjusted	0.54 (0.30–0.97)	0.0409	**0.63 (0.24–1.66)**	**0.3483**
**Mortality - Hispanic/Latino**				
Crude	0.45 (0.31 - 0.66)	<0.0001	**0.76 (0.40 - 1.45)**	**0.4057**
Adjusted	0.53 (0.35–0.81)	0.0029	**0.92 (0.47–1.83)**	**0.8195**
**Mortality - White**				
Crude	0.47 (0.37 - 0.60)	<0.0001	0.39 (0.28 - 0.55)	<0.0001
Adjusted	0.60 (0.47–0.78)	0.0001	0.50 (0.35–0.72)	0.0001
**Readmission - Asian/Pacific Islanders**				
Crude	0.56 (0.41 - 0.75)	0.0001	0.53 (0.35 - 0.82)	0.0033
Adjusted	0.62 (0.44–0.88)	0.0067	**0.80 (0.50–1.27)**	**0.3466**
**Readmission - African American**				
Crude	0.54 (0.40 - 0.72)	<0.0001	0.64 (0.42 - 0.96)	0.0292
Adjusted	0.64 (0.47–0.89)	0.0073	0.55 (0.35–0.86)	0.0084
**Readmission - Hispanic/Latino**				
Crude	0.78 (0.62 - 0.97)	0.0236	0.69 (0.50 - 0.94)	0.0186
Adjusted	0.79 (0.62–1.00)	0.0477	**0.81 (0.58–1.12)**	**0.197**
**Readmission - White**				
Crude	0.61 (0.53 - 0.71)	<0.0001	0.66 (0.53 - 0.82)	0.0002
Adjusted	0.69 (0.59–0.80)	<0.0001	0.78 (0.62–0.98)	0.0324

**Note:** values that did not reach statistical significance are **bolded**.

“Crude” corresponds to data not adjusted using covariates and “Adjusted” corresponds to data adjusted for covariates using Cox model.

Abbreviations: HR = Hazard Ratio.

1 Log Rank p-Value.

HBCR graduates had a lower adjusted mortality hazard compared to non-graduates (HR 0.57, 95 % CI 0.43–0.75, *P* < 0.0001). With stratification by sex, mortality hazards in both sexes were also significantly reduced. In multivariate analysis, male graduates had significantly reduced mortality hazards. Female graduates also had reduced mortality hazards, though this was not significant. With stratification by race, mortality hazards were significantly reduced for Asian/Pacific Islander and White graduates. While African American and Hispanic/Latino graduates also had reduction of mortality hazard, it was not significant. In multivariate analysis, only White graduates had significantly reduced mortality hazard. Graduates of all other races had reduced mortality hazards, but not significant (Table 2).

HBCR graduates had a lower adjusted hazard for all-cause hospital readmission compared to non-enrolled patients (HR 0.70, 95 % CI 0.62–0.78, *P* < 0.0001). With stratification by sex, readmission hazards in graduates of both sexes were also significantly reduced. The results remained unchanged in multivariate analysis. With stratification by race, readmission hazards in graduates of all races were also significantly reduced. In multivariate analysis, readmission hazards were significantly reduced for Asian/Pacific Islander, African American, and White graduates. Hispanic/Latino graduates had reduced readmission hazard, but not significant (Table 2).

HBCR graduates had a lower adjusted hazard for all-cause hospital readmission compared to non-graduates (HR 0.73, 95 % CI 0.62–0.85, *P* < 0.0001). With stratification by sex, readmission hazards in graduates of both sexes were also significantly reduced. The results remained the same in multivariate analysis. With stratification by race, readmission hazards in graduates of all races were also significantly reduced. In multivariate analysis, readmission hazards were significantly reduced for African American and White graduates. Asian/Pacific Islander and Hispanic/Latino graduates also had reduced readmission hazards, but they were not significant (Table 2).

Compared to the above multivariate analyses, the sensitivity analysis using the propensity adjustment method yielded similar findings.

**Fig. 2:**
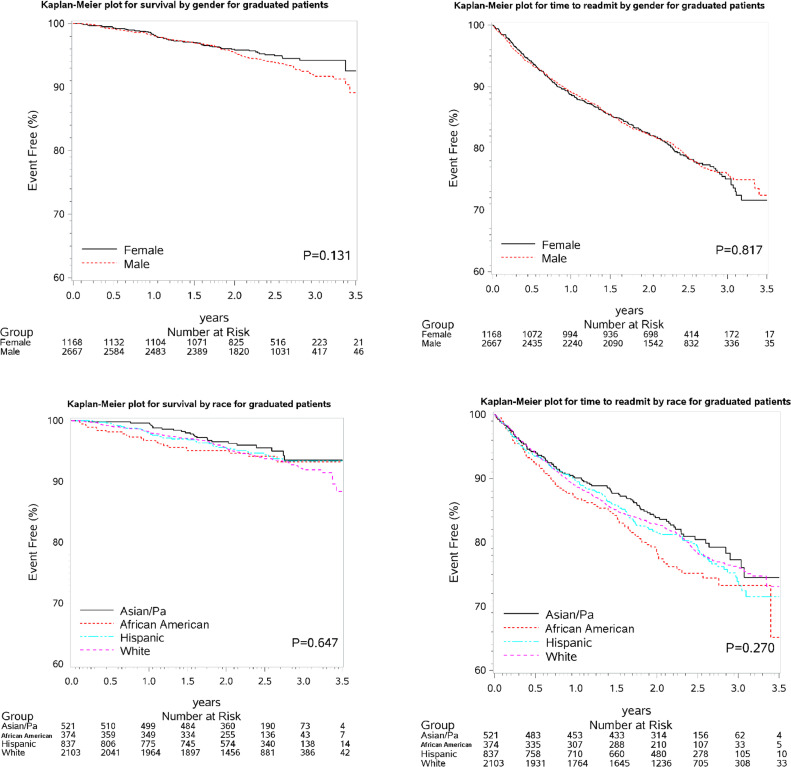
Kaplan-meier curves for survival and all-cause hospital readmission of HBCR graduates. Abbreviations: Pa = Pacific Islander.

Among HBCR graduates, mortality hazards did not differ significantly between males and females (*P* = 0.1306). Mortality hazards also did not differ significantly among Asian/Pacific Islanders, African American, Hispanic/Latino, and White (*P* = 0.6468). Similar patterns were observed for hazard of all-cause hospital readmissions, with no significant difference between sexes (*P* = 0.8168). Readmission hazards also did not differ significantly among races (*P* = 0.2705) (Fig. 2).

## 4 Discussion

In our survival analysis of 6868 patients referred to HBCR at KPSC, we observed that HBCR graduates had significantly lower hazards of all-cause mortality and hospital readmission compared to their non-enrolled counterparts. As expected, these findings are congruent with existing research on the effectiveness of HBCR in the reduction of mortality and hospital readmissions [5,15]. Notably, when we stratified the data by sex and race, these findings remained true in both crude and multivariate analyses.

A review of prior major CR observational studies with mixed indications revealed reductions in all-cause mortality among CR participants. Notably, a large 2020 Dutch CBCR study involving 83.687 patients with a mean follow-up duration of 1.8 years showed significant reduction in all-cause mortality among participants compared to control (adjusted HR 0.68, 95 % CI 0.65–0.71) [23]. Concordantly, a recent 2023 U.S. HBCR study with 1120 patients and a median follow-up duration of 4.2 years also showed significant reduction in all-cause mortality among participants compared to control (HR 0.64, 95 % CI 0.45–0.90, *P* = 0.01) [24]. Our study corroborates these findings, exhibiting a greater, yet comparable reduction in all-cause mortality among HBCR graduates (HR 0.57, 95 % CI 0.47–0.69, *P* < 0.0001). Similarly, significant reductions in hospital readmission among CR participants have also been demonstrated by prior studies. However, it is noteworthy that these studies included patients referred specifically for CAD, in contrast to our mixed indications [1,25,26].

In comparison to HBCR non-graduates, the graduates also demonstrated trends of lower hazards for all-cause mortality and hospital readmission, although not all comparisons reached statistical significance (Table 2). This may be attributable to the smaller sample size of HBCR non-graduates. In addition, there is significant variation in the degree of HBCR participation among non-graduates.

A review of existing literature demonstrated dose-dependent benefits of CR. A 2017 meta-analysis incorporated 33 randomized or non-randomized trials, wherein patients participated in CBCR, HBCR, or hybrid CR programs, stratified CR dosing into low (4–11 sessions), medium (12–35 sessions), and high (≥ 36 sessions) categories. Compared to recipients of low-dose CR, greater all-cause mortality reductions were observed among recipients of medium- or high-dose CR. Interestingly, no association between CR dose and all-cause hospitalization was observed [27]. Overall, these findings were consistent with our conclusions that greater HBCR participation, as exemplified by program graduation, conferred more benefits. The dose-response observations of CR were also reported in other studies, although these studies included only patients with CAD as indications for CR [28,29].

Our study has several notable strengths. To the best of our knowledge, this is the largest published retrospective cohort analysis on HBCR to date. The population of patients included in this study is diverse in many aspects, particularly in terms of demographics. Many randomized controlled trials (RCTs) and observational studies on HBCR have had limited sex diversity, with a low proportion of, and sometimes no female participants [5,14,[30], [31], [32], [33], [34]]. Racial diversity was also limited, and some of the studies did not report racial information [5,14,[30], [31], [32], [33], [34], [35]]. In addition, our study population included higher-risk patients with numerous comorbidities. Most previous studies on HBCR enlisted low-to-moderate risk patients, making it challenging to draw conclusions for higher-risk patients [5]. The primary and secondary endpoints of our study are all-cause mortality and hospital readmission, respectively, which are key patient outcome measures. Many studies in the literature do not include mortality and/or hospital readmission as either a primary or secondary endpoint [14,31,[33], [34], [35]]. Our study has an average follow-up period of 2.28 years, which exceeds most published studies on HBCR investigating mortality and hospital readmission [5,14,30,31,[33], [34], [35]]. This allows us to gain valuable insights into the long-term effects of HBCR.

Nonetheless, our study has limitations. Although all patients in the study had at least one indication for HBCR, the decision to refer them to HBCR was ultimately made by the treating physician, potentially introducing biases due to the non-randomized referral process. When deciding on referrals to either CBCR or HBCR, various individual circumstances, particularly accessibility, are considered. At KPSC, the general policy is to refer patients exclusively to either CBCR or HBCR without crossover. This study focused on HBCR, and we did not have access to CBCR patient data due to Health Insurance Portability and Accountability Act (HIPAA) regulations, which made it challenging to entirely rule out the possibility of crossovers. Furthermore, patients may independently choose to engage in extra exercise or rehabilitation activities outside of KPSC, introducing another potential source of bias. In addition, the study was conducted within KPSC, a large integrated healthcare system; and therefore, the generalizability of the study's findings to other healthcare settings may be limited. In the future, studies featuring patients outside an integrated healthcare system and studies that investigate different models of HBCR with varying intensities could help better optimize HBCR as a model for secondary prevention to improve cardiovascular health outcomes. Another limitation is the use of TAV attendance as a metric to determine participation/graduation status. During the TAVs, HBCR nursing staff routine inquired about patient's exercise, nutrition, and medication adherence. These visits were also opportunities for encouragement, education, and feedback. Although the number of TAVs provides an objective measure of engagement, it does not offer detailed insights into individual patient progress. Lastly, while our analysis incorporated a multivariate Cox proportional hazard model to address biases from variations in baseline characteristics among groups, potential biases cannot be completely eliminated.

Our findings suggest that HBCR holds great potential to be an effective tool for secondary prevention while highlighting its capability to diminish disparities in cardiovascular health outcomes between sexes and among races. By facilitating rehabilitation in the home setting, HBCR mitigates barriers such as transportation and financial constraints, which are particularly pronounced in women and racial minorities who are disproportionately affected by adverse cardiovascular outcomes [[36], [37], [38]]. The accessibility of HBCR fills the gap by offering an alternative to those who might otherwise forgo rehabilitation due to barriers of access. Future research, particularly well-designed large RCTs with extended follow-up periods, could help ascertain the extent to which HBCR can serve as a suitable substitute for CBCR. Additionally, investigation of appropriate rehabilitation dosing for various patient profiles, identification of key parameters for monitoring, and exploration of effective monitoring mechanisms are important aspects of HBCR that warrant further exploration.

## 5 Conclusion

Our analysis suggests that successful completion of an HBCR program is associated with reductions of all-cause mortality and hospital readmissions across sexes and races. Additionally, partial fulfillment of HBCR is associated with risk reduction in some, but not all groups of patients. These findings highlight HBCR's potential as a valuable tool for secondary cardiovascular prevention. Proper implementation of HBCR can be pivotal in enhancing healthcare equity. Future guidelines and healthcare policies should advocate for the appropriate utilization and broader adoption of HBCR to benefit patients from diverse backgrounds. Further research is needed to solidify HBCR's role as an effective and inclusive modality for cardiovascular rehabilitation.

## Funding

This project received no funding.

## CRediT authorship contribution statement

**Zhengran Wang:** Writing – original draft, Methodology, Investigation, Conceptualization. **Rachid Elkoustaf:** Writing – review & editing, Supervision, Project administration, Investigation, Conceptualization. **Columbus Batiste:** Writing – review & editing, Supervision, Project administration. **Debora Lahti:** Writing – review & editing, Resources. **Janis F. Yao:** Writing – review & editing, Visualization, Formal analysis, Data curation. **Tadashi Funahashi:** Writing – review & editing, Supervision.

## Declaration of competing interest

The authors declare that they have no known competing financial interests or personal relationships that could have appeared to influence the work reported in this paper.
